# Controversies and Opportunities in the Use of Inflammatory Markers for Diagnosis or Risk Prediction in Fatty Liver Disease

**DOI:** 10.3389/fimmu.2020.634409

**Published:** 2021-02-09

**Authors:** Joeri Lambrecht, Frank Tacke

**Affiliations:** Department of Hepatology and Gastroenterology, Charité University Medicine Berlin, Berlin, Germany

**Keywords:** chronic liver disease, NAFLD, NASH, inflammation, biomarker, liquid biopsy

## Abstract

In the Western society, non-alcoholic fatty liver disease (NAFLD), characterized by the excessive accumulation of fat in the liver, represents the most common cause of chronic liver disease. If left untreated, approximately 15%–20% of patients with NAFLD will progress to non-alcoholic steatohepatitis (NASH), in which lobular inflammation, hepatocyte ballooning and fibrogenesis further contribute to a distorted liver architecture and function. NASH initiation has significant effects on liver-related mortality, as even the presence of early stage fibrosis increases the chances of adverse patient outcome. Therefore, adequate diagnostic tools for NASH are needed, to ensure that relevant therapeutic actions can be taken as soon as necessary. To date, the diagnostic gold standard remains the invasive liver biopsy, which is associated with several drawbacks such as high financial costs, procedural risks, and inter/intra-observer variability in histology analysis. As liver inflammation is a major hallmark of disease progression, inflammation-related circulating markers may represent an interesting source of non-invasive biomarkers for NAFLD/NASH. Examples for such markers include cytokines, chemokines or shed receptors from immune cells, circulating exosomes related to inflammation, and changing proportions of peripheral blood mononuclear cell (PBMC) subtypes. This review aims at documenting and critically discussing the utility of such novel inflammatory markers for NAFLD/NASH-diagnosis, patient stratification and risk prediction.

## Introduction

To date, non-alcoholic fatty liver disease (NAFLD) is the leading cause of chronic liver disease worldwide. It is a very heterogeneous disorder, that finds its aetiology in a complex and multifactorial interplay of different parameters, such as obesity, a sedentary life-style, the composition of the main nutrient-intake, insulin resistance, diabetes, alterations in the gut microbiome, and genetic predisposition (1). It mainly manifests as an excess fat disposition in the liver (≥5% hepatic steatosis), independent of injury or inflammation, a condition termed isolated steatosis or non-alcoholic fatty liver (NAFL). When not intervened, NAFL may progress to non-alcoholic steatohepatitis (NASH), which is characterised by the presence of injury, hepatocyte ballooning, inflammation, and a varying extent of fibrosis, and which enhances the risk of cirrhosis and hepatocellular carcinoma (HCC) development, thus strongly influencing liver-related mortality (2). Due to the current epidemic in excess body weight and the aging – and thus more diabetes-susceptible - world population, the NAFLD pathology will only increase further into modern society. Indeed, while in 2016, in the USA alone, the number of NAFLD patients clocked at 85.3 million, mathematical models estimate a prevalence of 100.9 million cases in 2030, a substantial increase of 18%. The numbers of patients suffering from the more severe NASH are thought to display an even more steep increase of 56%, going from 17.3 million patients in 2016 to an estimated 27 million in 2030. Subsequently, significant increases in the number of patients suffering from decompensated cirrhosis, HCC, and liver-related deaths will be observed (3).

Although the severity of NAFLD-prevalence and potential critical outcome, no efficient specific pharmacological interventions are currently available, besides the obligatory changes in lifestyle. Multiple clinical trials, however, are ongoing, evaluating the potential efficacy of novel pharmacotherapeutics in NAFLD patients (4). As the presence and severity of fibrosis has been identified as the major indicator of poor long-term outcome, including death and the need for a liver biopsy, in NASH patients (5, 6), it has become a critical determinant for the inclusion of NAFLD patients in clinical trials (7). Moreover, therapeutically obtained fibrosis regression is found to be closely correlated to the resolution of steatohepatitis and improvement of the NAFLD activity score (NAS) (8, 9), therefore highlighting the importance of evaluating the different NASH parameters for assessment of disease improvement. An accurate and efficient diagnostic tool for NAFLD evaluation is thus crucial to ensure a timely trial-inclusion of at-risk patients as well as subjecting these high-risk patients to more intense lifestyle interventions and surveillance for disease-related complications (10).

## Current Clinical Diagnostic Means for Fatty Liver Disease

At present, the liver biopsy, either percutaneous or transjugular, remains the gold standard for disease evaluation in patients with NAFLD. This invasive procedure is however associated with multiple drawbacks, including the limited representation of the total liver mass, intra- and interobserver variability, pain and post-procedure complications, and an extensive financial cost causing a subsequent doubtable cost-effectiveness ratio (11). The poor inter-reader variability was recently stressed by a secondary analysis from a (negative) clinical trial, in which 678 paired biopsies from 339 patients were independently read by three hepatopathologists. Particularly, the scoring for histological NASH, as well as treatment responses (NASH resolution, fibrosis improvement) showed a low inter-reader reliability (12). In addition, the high financial cost and the potential procedural risks, withhold the liver biopsy to be used as a tool for regular patient follow-up in medical practice, creating a severe hiatus in NAFLD-management. Multiple non-invasive techniques have therefore been suggested to be more suitable as manner of first-line investigation, including laboratory (blood)tests, and imaging-based tools (13).

Blood-based markers can be divided into direct markers, representing ECM production and degradation (hyaluronic acid, matrix metalloproteases, etc.), and indirect markers which indicate liver function and inflammation (aminotransferases, platelet counts, etc.) (11). Such circulating markers are often combined with risk characteristics (diabetes, age, etc.) into diagnostic scores to obtain maximum sensitivity and specificity. One popular example comprises the NAFLD fibrosis score (NFS), which includes age, aminotransferases, albumin, body mass index (BMI), platelet count, and glucose intolerance status, and which has a high negative predictive value for excluding NAFLD-patients with advanced fibrosis (14). Examples of other serological scoring tools are Fib-4, AST-to-platelet ratio index (APRI), Fibrotest, and enhanced liver fibrosis test (ELF). A recent meta-analysis, including 64 studies with a total of 13,046 NAFLD patients, compared the diagnostic performance of APRI, Fib-4, BARD score, and NFS for identification of advanced fibrosis, and reported the respective summary AUROCs of 0.77, 0.84, 0.76, and 0.84. This study concluded that Fib-4 and NFS have, of the analysed serological scoring tools, the highest accuracy and negative predictive value for ruling out advanced fibrosis (15). Moreover, as Fib-4 and NFS show the highest extent of validation in different NAFLD populations, with consistent accuracy in excluding advanced fibrosis (generating negative predictive values of over 90%), these scores are proposed as first-line screening method in clinical settings where more advanced/expensive tests are unavailable (16).

In the process of chronic liver damage, the liver acquires a stiffened character, due to the excessive deposition and accumulation of ECM by activated myofibroblasts. Such important change in elasticity may be visualized and quantified using different imaging-tools, having each their own accuracy, methodology, handling and interpretation (17). Especially the evaluation of liver stiffness through abdominal ultrasound has found its way into clinical practice, and comprises acoustic radiation force impulse (ARFI), (point) shear wave elastography, and (vibration-controlled) transient elastography (TE; Fibroscan), this latter being the most popular technique in current clinical practice (17). However, although specific adaptions (such as the development of an XL-probe) increase the utility of TE in patients with central obesity, excessive fat accumulation may still bear a risk of technical failure (18). One important advantage of TE is its potential to simultaneously evaluate the hepatic fat content through the additional feature of controlled attenuation parameter (CAP) (19). Furthermore, the combination of liver stiffness and CAP measures by TE with serological markers such as AST (FibroScan-AST/FAST score) can increase its accuracy in identifying patients at risk for progressive NASH (20).

Magnetic resonance imaging-based techniques, so called magnetic resonance elastography (MRE), represent another method of liver stiffness evaluation. MRE-results are less influenced by the presence of obesity, and may also simultaneously evaluate the extent of fibrosis and steatosis, the latter through use of the proton density fat fraction (PDFF), of which the accuracy has been suggested to be even higher than CAP (21). However, while MRI-based scoring tools have greater potential of correctly identifying the extent of steatosis and fibrosis in the affected liver, as compared to ultrasound-based techniques, due to their higher costs and low availability, they are more predominantly used in a research setting (22).

The above mentioned non-invasive diagnostic tools, are useful to narrow down the indication for liver biopsy, and help decide on the further clinical management of the patient. For example, in individuals suffering from obesity, who do not present any other feature of the metabolic syndrome, who have an APRI score ≤ 0.5, a FIB-4 score ≤ 1.1; and TE ≤ 6 kPa, the likelihood for the presence of significant liver fibrosis is very low, subsequently leading to a small liver-related mortality in the timeframe of 10 years (23, 24). In these cases, lifestyle changes combined with patient follow-up through use of stiffness measurement, are the best approach (2). However, in those patients with aberrant scoring values, the use of liver biopsy remains the reference standard for confirmation and evaluation of the presence of significant fibrosis (2).

Overall, the current non-invasive scoring tools lack sufficient sensitivity and specificity, especially for the early stages of liver disease, to make the liver biopsy completely aberrant. Additionally, almost no data is available on their usefulness in clinical follow-up. Therefore, novel diagnostic strategies are considered, focussing not only on the extent of fibrosis or damage of the affected liver, but also on liver inflammation, one other crucial aspect of liver pathogenesis.

## Inflammatory Processes During NASH

**Figure 1:**
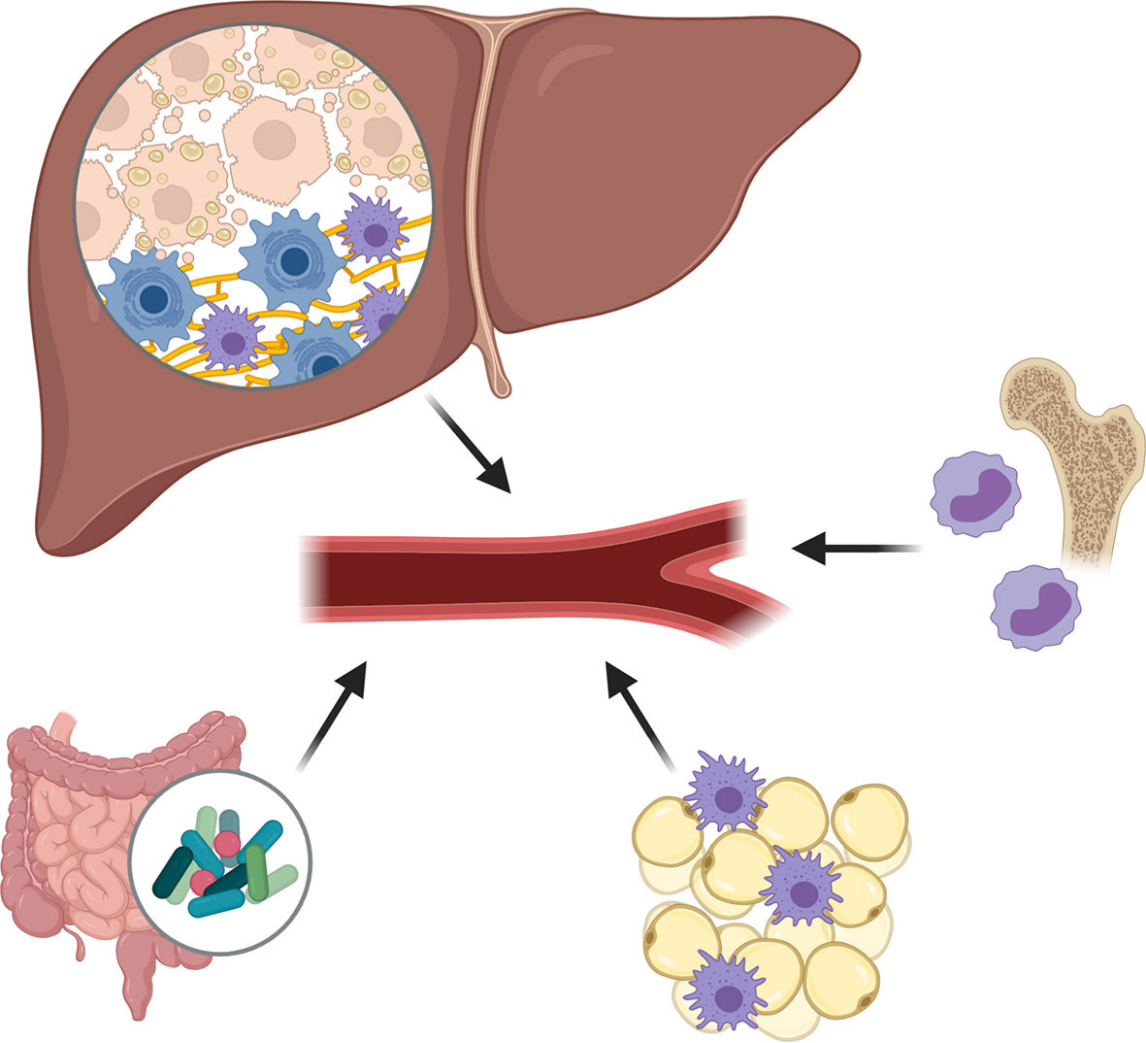
Damaging hits during non-alcoholic fatty liver disease (NAFLD) pathogenesis. The pathogenesis of NAFLD relies on multiple damaging “hits”, including the proliferation, dysfunction and inflammation of adipose tissue, the excessive presence of fatty acids and cholesterol in the liver and circulation, and important changes in gut permeability and microbiota composition causing an increased release of pathogen-associated molecular patterns. Subsequent to these damaging factors, an enhanced production and release of pro-inflammatory cytokines and chemokines is observed, creating a pro-inflammatory environment in which Kupffer cells become activated and monocyte-derived macrophages (MoMФs) and other leukocytes are recruited to the liver.

The pathogenesis of NAFLD relies on multiple damaging “hits” **(** **Figure 1** **)**, with the proliferation, dysfunction and inflammation of adipose tissue being one of them (25, 26). Indeed, in the visceral adipose tissue of NASH patients, an increased presence of, among other, CD11c+CD206 and CCR2+ macrophages can be found (27). Similar changes of intrahepatic accumulation of CCR2+ macrophages are present in the liver, particularly in patients with NASH-fibrosis (28). Such immune cell infiltration is accompanied by an increased release of chemokines and proinflammatory cytokines into the circulation, which have proven propagating effects on liver disease and insulin resistance (the so called adipose tissue – liver axis), and which serve as fuel for local and systemic inflammation (25, 26, 29).

One other liver damaging “hit” concerns the excessive presence of fatty acids (FAs) and cholesterol in the liver and circulation (30). While during normal liver homeostasis, FAs are metabolized by the hepatocytes to form triglycerides, during NAFLD progression, an excessive amount of FAs, impaired beta-oxidation, and diminished export of triglycerides as very low-density lipoproteins can be observed, causing an accumulation of saturated FAs and oxidized cholesterol in the hepatocytes. The subsequent formation of lipotoxic species, and endoplasmic reticulum- and oxidative-stress, leads to profound hepatocyte damage, which may eventually cause the hepatocytes to undergo necrosis, apoptosis, or necroptosis. Such hepatocyte death is associated with the release of reactive oxygen species and their cellular content, including nuclear and mitochondrial DNA, purine nucleotides (ATP, UTP) and other damage-associated molecular patterns (DAMPs) (30, 31). These metabolic danger signals may activate a plethora of pattern recognition receptors (PRRs) expressed by various liver cells, and which will trigger the production and release of pro-inflammatory cytokines and chemokines, creating a pro-inflammatory environment in which Kupffer cells become activated and monocyte-derived macrophages (MoMФs) and other leukocytes are recruited. This recruitment of pro-inflammatory cells, which significantly changes the immune cell composition of the liver during NAFLD, may occur *via* various chemoattractant-axes, including the CCL2/CCR2, CCL1/CCR8, CXCR6/CXCL16, and CCL25/CCR9-pathways, with the chemoattractants being secreted by activated Kupffer cells, liver sinusoidal endothelial cells, and hepatic stellate cells. Besides the enhanced recruitment, also an enhanced polarization of macrophages toward a pro-inflammatory (“M1-like”) phenotype can be observed, potentially caused by stimulating cytokines such as TNF and IFN-γ (30, 32).

The major changes in gut permeability and microbiota composition, form an additional plausible harmful “hit”. Multiple alterations can be observed that range from reduced microbial diversity, abundance of pathogenic microbiota, accumulation of bacterial metabolites, changes to the intestinal virome and reduced intestinal barrier (33, 34). With these changes, an increased release of pathogen-associated molecular patterns (PAMPs), e.g., lipopolysaccharide (LPS) can be observed in the portal vein and the systemic circulation, which stimulate the respective PRRs expressed by the various liver cells, and therefore further contribute to the pro-inflammatory liver environment (35).

Many experimental studies revealed that the “sterile” inflammation observed during NAFLD leads to a perpetuation of liver disease. Indeed, studies depleting certain types of immune cells (36, 37), or blocking the polarization and recruitment of inflammatory cells, have led to significant alleviation of fibrosis in various mouse models and early-stage clinical trials (32). However, it should not be forgotten that the inflammatory response is also crucial for healing and tissue repair, often observed during the early stages of liver injury (38). While this dual function of the inflammatory system may have a more hampering effect on its use as therapeutic target, the overall presence of inflammation may represent an interesting tool for the diagnosis and follow-up of disease **(** **Table 1** **)**. Therefore, next, we will describe and discuss potential new inflammatory markers for NAFLD-evaluation.

**Table 1:** Selected experimental inflammatory biomarkers in NAFLD.

Biomarker	Predominant Cellular source	Application	Conflicting results reported?	Strength of evidence
**(high sensitivity) C-reactive protein**	Hepatocytes, macrophages, lymphocytes	- NASH vs NAFL- NAFLD severity- prediction cardiovascular complications	Yes, by multiple studies. CRP seems strongly influenced by body fat.	+/-
**Pentraxin 3**	Neutrophils, macrophages, monocytes, dendritic cells	- NAFLD vs controls- NASH vs NAFL- NAFLD severity	Yes, 1 study reported the inability of pentraxin 3 to identify NASH	+
**Interleukins**				
*IL-1β*	Monocytes, macrophages	- NASH vs NAFL	No	++
*IL-6*	Monocytes, macrophages	- NAFLD vs controls- NASH vs NAFL- advanced fibrosis vs no or mild fibrosis	No	+++
*IL-8*	Monocytes, macrophages	- NAFLD vs controls- correlation to stage of fibrosis- correlation to NAFLD severity	No	+++
*IL-12*	Dendritic cells, monocytes, macrophages	- NAFLD severity	No	+ (only 1 study)
*IL-32*	NK cells, monocytes, T-cells	- NAFLD vs controls- NASH vs NAFL	No	+ (only 1 study)
**Lipocalin-2**	Neutrophils, hepatocytes	- NAFLD vs controls- NASH vs NAFL	No	+++
**TNFα**	Macrophages, NK cells, lymphocytes	- NAFLD vs controls	Yes, disputed correlation with severity of NAFLD	+
**sTNFR1/2**	Large variety of cell types	- NASH vs NAFL- advanced fibrosis vs no or mild fibrosis- Paediatric NAFLD vs controls- Paediatric NASH vs NAFL	No	+++
**sCD14**	Monocytes, macrophages	- NASH vs NAFL- correlation to NAS-score	Yes, disputed possibility to indicate NAFLD/NASH	+
**sCD36**	Macrophages, hepatocytes	- NAFLD vs controls- correlation to NAFLD severity	No	+++
**sCD163**	Monocytes, macrophages	- NAFLD vs controls- NASH vs NAFL- NAFLD severity- advanced fibrosis vs no or mild fibrosis- Treatment response- Paediatric NAFLD vs controls	No	++++
**Extracellular vesicle-phenotypes**				
*CD14^+^*	Monocytes, macrophages	- NAFLD vs controls- NAFLD severity	No	+ (only 1 study)
*Vα21/Vβ11^+^*	iNKT-cells	- NAFLD vs controls- NAFLD severity	No	+ (only 1 study)
*CD14^+^/CD16^+^*	Monocytes, macrophages	- advanced fibrosis vs no or mild fibrosis	No	+ (only 1 study)
**Quantity immune cell subtypes & their phenotypes**				
*↑ CD14^+^CD16^++^ monocytes ↓ CD14^++^CD16^-^ monocytes*	N.A.	- NAFLD vs controls	No	++
*CCR4 expression on monocytes*	N.A.	- NAFLD vs controls	No	+ (only 1 study)
*TLR6 expression on monocytes*	N.A.	- NAFLD vs controls- NASH vs NAFL	No	+ (only 1 study)
*CD25^+^CD45RA^+^CD4^+^ T-cells*	N.A.	- NAFLD vs controls- Significant fibrosis vs no or mild fibrosis	No	+ (only 1 study)
*CXCR3^+^CD4^+^ T-cells*	N.A.	- NAFLD vs controls- Significant fibrosis vs no or mild fibrosis	No	+ (only 1 study)
*PD1^+^CD25^+^CD45RA^+^CD4^+^ T-cells*	N.A.	- NAFLD vs controls- Significant fibrosis vs no or mild fibrosis	No	+ (only 1 study)
*Th17/rTreg*	N.A.	- NASH vs NAFL	No	+ (only 1 study)
*Th2/rTreg*	N.A.	- NASH vs NAFL	No	+ (only 1 study)
*Neutrophil-to-lymphocyte ratio*	N.A.	- NASH vs NAFL- NAFLD severity- advanced fibrosis vs no or mild fibrosis- Liver disease related mortality	No	++++

References for the mentioned circulating markers can be found in the main text.

N.A., not applicable; NASH, non-alcoholic steatohepatitis; NAFLD, non-alcoholic fatty liver disease; NAFL, non-alcoholic fatty liver; IL, interleukin; NK, natural killer; TNFα, tumour necrosis factor alpha; sTNFR, soluble tumour necrosis factor receptor; CD, cluster of differentiation; iNKT, invariant natural killer; CCR, C-C chemokine receptor; TLR, toll-like receptor; CXCR, C-X-C chemokine receptor; PD1, programmed cell death protein 1; Th, T helper cell; rTreg, resting regulatory T cells.

## Inflammatory Markers

### C-Reactive Protein

During the acute phase of the inflammatory process associated with NAFLD development, an IL-6-dependent increased transcription and subsequent release of C-reactive protein (CRP) by the liver is observed. In the addition to the standard techniques for CRP evaluation (mainly through immunoassay), high-sensitivity CRP detection tests have been developed, which thus allow the detection of low-grade systemic inflammation. Various studies have identified elevated circulating high-sensitive (hs) CRP levels as indicators for histology- (39, 40) or ultrasound-based (41, 42) NASH. Elevated hsCRP levels were even found to be associated with the severity of NAFLD (43) and to distinguish patients with advanced fibrosis from those with mild fibrosis (40). This latter finding also correlated to the elevated CRP mRNA levels that were found in the liver tissue of NASH patients (40). Additionally, elevated hsCRP levels in NAFLD patients seem to have a predictive value for the development of cardiovascular complications (43). Such association between (hs)CRP levels and cardiovascular disease has already been proven outside the NAFLD pathology (44).

Although the previous mentioned studies claim the diagnostic utility of circulating hsCRP in NAFLD, results of other studies contradict this hypothesis, being unable to find any relationship between hsCRP and the extent of hepatic steatosis, necroinflammation, and fibrosis (45, 46), especially when hsCRP values are being corrected for visceral fat, BMI, gender or age of the patient (47, 48). Indeed, circulating hsCRP-levels are known to be associated with BMI, systolic blood-pressure, waist-to-hip ratio, insulin resistance, and concentrations of HDL cholesterol and triglycerides (49). Especially the amount of fat seems to be an important determinant of hsCRP levels, as it has been found that per 10% increase in BMI, circulating hsCRP levels increase by 19-20% (46).

### Pentraxin 3

Pentraxin 3 (PTX3) is a member of the long chain pentraxin family, like CRP, and is a marker of the acute phase inflammatory response (50). It is predominantly produced by immune cells, although other cells and tissues may also contribute to its production, as a response to pro-inflammatory signals. Various studies have identified the elevated presence of circulating PTX3 in patients with NAFLD, as compared to controls (51–53), in NASH patients as compared to patients with simple steatosis (51, 54) and in correlation with the severity of hepatic fibrosis (51, 54, 55). Additionally, PTX3 levels were associated with triglyceride concentrations, LDL-cholesterol, and waist circumference (53) and was found to be increased in obese children and adolescents with increasing severity of fatty liver (56). However, one study reports the inability to find any significant diagnostic value of PTX3 for the identification of NASH, nor any correlation with the severity of the disease (57), thus nuancing its diagnostic utility.

### Interleukins

Interleukins (ILs), a heterogeneous group of cytokines which play essential roles on the activation and differentiation of immune cells, have been shown to play essential roles in NAFLD pathogenesis, what makes them interesting therapeutic targets (58–60). Their utility in diagnosis of NAFLD has been evaluated in various studies, and identified that several specific interleukins possess significant sensitivity and specificity as stand-alone marker, or in combination with other biomarkers. We describe some of the interleukins with diagnostic potential.

Circulating IL-6 levels show significant value for the discrimination of NAFLD patients from obese individuals (61). Moreover, a progressive increase during the NAFLD pathogenesis is observed, as proven by the potential of circulating IL-6 levels to identify patients with simple steatosis (NAFL) to healthy controls (62), to identify patients with NASH as compared to those with simple steatosis (63), and to discriminate NASH patients with advanced fibrosis, from patients with no or mild fibrosis (64). Interestingly, also the expression of its receptor (IL-6R) has been found to be elevated in the circulation of NASH patients (63). The elevated circulating IL-6 levels in NASH patients were found to be associated with the hepatic IL-6 protein levels and the overall degree of hepatic inflammation and fibrosis (65). It should be noted that on mRNA-level, one other study found no difference in hepatic IL-6 expression between individuals with NASH and those with simple steatosis (40).

In contrary to IL-6, the hepatic mRNA-expression levels of IL-8 are significantly increased in NASH patients with advanced fibrosis. IL-8 is an important chemoattractant for neutrophils and possibly other myeloid immune cells (66). Such changing IL-8 levels are reflected in the serum of these NASH patients, as elevated circulating IL-8 levels are found to correlate to the stage of fibrosis (67). Moreover, IL-8 levels can identify the presence of significant fibrosis in NASH patients (68), and are elevated in NAFLD patients when compared to both obese and non-obese controls (61). Additionally, a positive correlation between circulating IL-8 levels and the histological NASH parameters, lobular inflammation and hepatocellular ballooning, was observed in NASH patients suffering from (morbid) obesity (68, 69). The importance of IL-8 as diagnostic tool is further demonstrated by the possibility to, as only parameter in a panel of 24 analysed cytokines, significantly correlate with hepatic fibrosis after controlling for age, sex, BMI, hypertension, metabolic syndrome, and diabetes mellitus (67).

IL-12 is a pro-inflammatory cytokine, known for its aggravating effect on liver disease, through the induction of a T-helper (Th)-1 phenotype in Th cells and inhibition of the Th-2 phenotype (70). An elevation of circulating IL-12 levels is associated with NAFLD-severity, as observed in an ultrasound-staged NAFLD population of 100 individuals (71).

Transcriptomics analysis on hepatic tissue identified up-regulated IL-32 levels in NAFLD patients, as compared to liver tissue obtained from obese individuals showing no signs of hepatic steatosis. Such elevated IL-32 mRNA levels were found to be associated with body mass index (BMI), aminotransferases, NAFLD activity score, and homeostasis model assessment of insulin resistance (HOMA-IR) index (72). Another transcriptomics-based study, including NAFLD patients with and without the PNPLA3 I148M genetic risk variant, identified a similar robust up-regulation of IL-32 in individuals with a severe phenotype (defined as NAFLD activity score ≥ 4, fibrosis stage ≥ 2, or presence of steatohepatitis), independently of the underlying genotype. The evaluation of circulating IL-32 levels identified its potential to diagnose NAFLD, and development of NASH. Surprisingly, these elevated levels were found to be independent of aminotransferases. No differences between PNPLA3 I148M carriers and non-carriers were observed (73).

The NAFLD pathology is strongly associated with inflammasome activation, with the nucleotide-binding oligomerization domain-like receptor pyrin domain containing 3 (NLRP3) inflammasome being the most extensively studied. In the presence of danger signals, the NLRP3 protein complex activates the protease caspase-1, which initiates the maturation of the pro-inflammatory cytokines IL-1β and IL-18. In the liver, the NLRP3-caspase-1 complex is predominantly expressed in KCs, but may also be present in parenchymal cells and other inflammatory cells (74). IL-1β plays a key role in liver disease, as it affects both steatosis, inflammation and fibrosis. Indeed, IL-1β stimulates triglyceride and cholesterol accumulation in hepatocytes, promotes the recruitment of immune cells through up-regulation of ICAM-1 on liver sinusoidal endothelial cells, and stimulates local inflammation through induction of IL-6 production (59). This strong association of IL-1β with disease severity is also reflected in the circulation, as elevated blood-levels of IL-1β are found in patients with NASH, compared to those with simple steatosis (75, 76). Interestingly, also elevated hepatic and circulating levels of IL-1 receptor antagonist (IL-1RA) are observed in NASH patients, and were even found to be correlated to the degree of lobular inflammation (77). While circulating IL-18 levels show a positive correlation with waist circumference, insulin resistance, the development of atherosclerosis, and triglyceride content in patients with metabolic syndrome (78), no changes in circulating expression levels were observed between NASH patients and patients with simple steatosis, or NAFLD patients and healthy individuals (79, 80). Remarkably, in mice receiving a “American lifestyle-induced obesity syndrome” diet, elevated levels of IL-18, but not IL-1β, were observed, in contrast to the above mentioned human data (81).

### Neutrophil-to-Lymphocyte Ratio

Neutrophils, protagonists of the innate immunity response, differentiate from myeloid precursors in the bone marrow, and are recruited into the blood stream through various signalling molecules such as granulocyte colony stimulating factor (G-CSF), CXC chemokine receptor (CXCR) 2 and 4 (82). Calculating the ratio of the number of neutrophils to lymphocytes (the neutrophil-to-lymphocyte ratio; NLR) harbors the potential to discriminate individuals with NASH from those with NAFL (83, 84). Indeed, using a cut-off value of 1.9, a sensitivity and specificity of respectively 72% and 70% is obtained for the identification of NASH-patients (85). Furthermore, NLR is able to discriminate NASH patients with advanced fibrosis, from those with mild-to-moderate fibrosis, indicating the significant association of NLR with the extent of histological features of NASH (83, 85). Additionally, due to the many effects of neutrophils on the immunological and metabolic features of NASH, NLR may also give information concerning hepatic steatosis, insulin resistance, hyperlipidaemia, imbalanced metabolic hormones, and abundance of pro-inflammatory signals (86). It is important to mention that NLR may also predict liver-disease related mortality. Indeed, analysis of the NLR in 570 patients with end-stage liver disease, of which 54 were NAFLD-patients, identified a significant correlation between increasing NLR and mortality within 3 months of listing for transplantation, and an overall association with high 3-month mortality when NLR ≥ 5 (87).

### Lipocalin-2

Lipocalin-2 (LCN2), also known as neutrophil gelatinase-associated lipocalin (NGAL), is a secretory glycoprotein, which has a pro-inflammatory action, and which is thought to be strongly involved in the metabolic and cardiovascular complications associated with obesity (88). An overall increase in circulating LCN2 is observed in NAFLD patients, as compared to healthy controls, which additionally correlated to insulin resistance and inflammation (this latter was evaluated through CRP) (89). One other study, which included biopsy-staged NAFLD patients, identified the elevated circulating LCN2 levels in patients with NASH, as compared to NAFL patients or healthy controls. Moreover, in all NAFLD patients, LCN2 levels positively correlated to the injury-associated markers ALT, AST, and γGT. Co-localization of LCN2 with CD66b, a general neutrophil marker, in the liver biopsies of NASH patients further indicated the neutrophilic origin of the circulating LCN2 (90). Other recent studies renounce this neutrophil-exclusive origin of LCN2, claiming that also hepatocytes, adipocytes, and endothelia contribute to LCN2 production and secretion (91, 92), therefore questioning its exclusive representation of liver inflammation.

### Tumour Necrosis Factor Alpha

One of the major pathogenic drivers of the NAFLD pathology is tumour necrosis factor alpha (TNF-α), which mediates liver injury mainly *via* TNF-receptor-1 (TNFR1) signalling (93). Indeed, blocking this signalling pathway through use of anti-TNF-α (infliximab) (94, 95) or anti-TNFR1 (96) antibodies in rodent models of NAFLD, led to significant improvement of liver steatosis, inflammation, insulin resistance and extent of liver fibrosis. Furthermore, the presence of the rs1799964 single nucleotide polymorphism (SNP) in the TNF risk allele has been found to be an independent risk factor for an enhanced rate of histological progression (97).

Besides its potential as a therapeutic target for treatment of NAFLD, its diagnostic utility has been widely discussed. In multiple studies, circulating levels of TNF-α have been found to identify the presence of NAFLD (98, 99), which would reflect up-regulated TNF-α mRNA expression in the affected liver (99). However, contradictory results concerning the sensitivity of circulating TNF-α levels for the identification of the different stages of the NAFLD pathology, have been reported. While some studies only report its potential to discriminate NASH-patients with cirrhosis from healthy subjects, being unable to show any correlation with the severity of the histopathology (100), others report its potential to distinguish NASH-patients with significant fibrosis from those with no or mild fibrosis (68).

Not only TNF-α itself, but also its soluble receptors (sTNFR1/2) have an increased presence in the blood stream of NASH patients, as compared to patients with simple steatosis (63, 99, 101). Furthermore, circulating levels of sTNFR-2 in NASH were even suggested to discriminate between advanced- and low stage-fibrosis. Interestingly, such sTNFR-2 were found to be higher in patients with diabetes mellitus (DM) compared to those without DM (102).

Lastly, circulating TNF-α and sTNFR1/2 levels might also have a diagnostic utility in paediatric NAFLD patients, as their levels were found to be increased in children with NAFLD (103) and NASH (104). However, these markers did not allow discrimination between advanced and no-mild liver steatosis (103).

### CD14

CD14 is a multifunctional receptor, with a constitutive expression on the cell surface of various immune cells. In the liver, different macrophage populations express CD14 (105). Its main function is the recognition of LPS, or other components of the bacterial wall, causing the activation of a plethora of signalling cascades, eventually leading to cytokine production and shedding of its extracellular domain (sCD14) (106, 107). During NAFLD development, a strong correlation between the presence of CD14-positive immune cells, and the extent of necroinflammation and fibrosis has been observed (108). Additionally, the C/T (-159) polymorphism in the CD14 gene (rs2569190) has been closely linked to an increased risk in the development of NAFLD, however, without however influencing the degree of hepatic steatosis or fibrosis (109).

Circulating levels of soluble CD14 (sCD14), which has been claimed to be mainly derived from the liver (110), were found to be inversely correlated to insulin resistance and markers of liver injury (ALT and γGT) in both lean and obese individuals (111, 112). Additionally, changing levels of sCD14 accompanied the significant changes in hepatic necro-inflammation, and overall NAS-score, in obese individuals undergoing surgically induced weight loss (113). In a cohort of 113 NAFLD patients; sCD14 levels showed diagnostic value for the presence of NASH, and a strong correlation to liver inflammation (114), thus suggesting its diagnostic utility in this pathology. One other study, however, questions these results, as they only found such dynamic sCD14 levels in obese patients, as compared to healthy individuals, but not in relation to the development or severity of NAFLD/NASH (115).

### CD36

One important example of a scavenger receptor, a pattern-recognition receptor, on phagocytic cells concerns CD36. Indeed, this receptor recognizes a plethora of both foreign material, such as lipids and lipoprotein components of bacterial cell walls, and endogenously derived ligands, including oxidized phospholipids, glycated proteins, and apoptotic cells. As activation of the CD36 causes the induction of pro-inflammatory signalling, its contribution to the development of inflammatory disease is evident (116). Besides macrophages, the presence of CD36 has also been identified on adipocytes, myocytes, enterocytes, and hepatocytes, where it regulates free fatty acid (FFA) transport and oxidation, VLDL secretion, and autophagy (117). Moreover, its stimulating effect on FFA uptake caused the CD36 receptor to act as driving force of the initiation and perpetuation of liver steatosis (118), thus displaying key functions in the different aspects of NAFLD pathogenesis.

The importance of CD36 in metabolic liver disease has been suggested by the close correlation between hepatic CD36 mRNA and protein expression and the liver fat content in morbidly obese patients (119, 120), and by its elevated presence in the livers of NAFLD patients compared to healthy controls (121, 122). This enhanced hepatic presence is represented in the blood-stream, as soluble CD36 (sCD36) was found to be increased in biopsy-staged NAFLD patients, compared to healthy controls, and as the circulating levels were even correlated with the histological grade of steatosis (123). sCD36 can also nicely distinguish patients with simple steatosis from healthy individuals (123, 124), and would correlate well with the extent of intrahepatic lipids (as measured by magnetic resonance spectroscopy) in NAFLD patients, therefore suggesting that this circulating marker especially represents the metabolic aspect of NAFLD pathology, and less of the immunological aspects. Lastly, it should be mentioned that sCD36 is tightly correlated to the presence of insulin resistance in obese patients with type 2 diabetes mellitus (125, 126), further suggesting its -especially- metabolic representation.

### CD163

CD163 is a scavenger receptor, which has as main function the recognition of the tight complex of haptoglobin and haemoglobin, known to be formed after red blood cell haemolysis. While both monocytes and macrophages express CD163, its expression especially becomes elevated during macrophage-maturation (127, 128). The presence of a soluble form of CD163, sCD163, has been observed in the bloodstream, and is thought to be mainly derived from proteolytic shedding in response to various inflammatory responses such as LPS, oxidative stress and thrombin (128, 129). A significant portion of sCD163 is potentially derived from Kupffer cells, as sCD163-concentrations are found to be 23% higher in the hepatic vein, as compared to the portal vein, in patients with obesity or NAFLD (130). Interestingly, no differences in sCD163 concentrations were observed between the portal and hepatic vein in healthy individuals (131).

In adult NAFLD patients, circulating levels of sCD163 are elevated, when compared to healthy controls. Furthermore, sCD163 has been described as able to discriminate NASH from simple steatosis, and to correlate with the extent of steatosis, inflammation, and hepatocellular ballooning (132). Indeed, other studies confirmed the close correlation of sCD163 with the histological extent of NAFLD (133), its utility for identification of NAFLD-induced advanced fibrosis (134), and its association with markers of liver necro-inflammation and glucose-homeostasis (135). sCD163 might also reflect treatment response, as decreasing circulating expression levels were observed in obese patients undergoing bariatric surgery (130), and NAFLD patients undergoing life-style intervention (135, 136)

Also in paediatric NAFLD cases, sCD163 may have diagnostic utility. Indeed, in children with biopsy-proven NAFLD or NASH, a significant increase in the amount of hepatic CD163-positive cells was observed in those with severe histological activity, and in close correlation with the presence and extent of fibrosis (137). Furthermore, in obese children, an elevated presence of sCD163 is observed in those with ultrasonographic-proven steatosis and elevated transaminase-levels (138).

### Extracellular Vesicles

Extracellular vesicles (EVs) are small, membrane-derived structures which can be divided into three subtypes (exosomes, microvesicles, and apoptotic bodies), and are released by the cells into their microenvironment. The EV-cargo may consist of messenger RNA (mRNA), micro-RNA (miRNA), long non-coding RNA (lncRNA), lipids, and proteins, and strongly reflects the cytosolic and membrane composition of its cell of origin, therefore suggesting the use of blood-circulating EVs to represent disease-associated cellular changes (11). Besides such changing cargo, also changes in the absolute numbers of circulating EVs may be observed during disease. For example, an elevated number of circulating EVs is observed in NASH patients, with the EV-numbers strongly correlating to NASH clinical characteristics and disease severity (139). These findings reflect the elevated number of circulating EVs identified in mice with diet-induced NASH (140). Moreover, while in these mouse models the circulating hepatocyte-derived (ASGR1^+^ and CYP2E1^+^) EVs were found to be already increased after 12 weeks of feeding, macrophage (Galectin 3^+^)- and neutrophil (Ly-6G and Ly-6C)- derived EV were only elevated after 48 weeks of feeding, and were associated with the histological presence of inflammatory foci in the liver (140). These results suggest that circulating extracellular vesicles derived from specific subsets of inflammatory cells might have significant diagnostic utility. Such EV analysis in human patients identified the potential of CD14^+^ EVs, derived from monocytes and macrophages, and Vα21/Vβ11^+^ EVs, derived from invariant NK T (iNKT)-cells, to diagnose patients with NAFLD from healthy individuals. Furthermore, quantities of these specific EV-subsets were correlated to ALT levels and the overall severity of NASH (141). Although CD4^+^ and CD8^+^ EVs were also up-regulated in NAFLD individuals, they displayed less diagnostic utility for the extent of steatosis (141). Another study identified changing numbers of CD14^+^ and CD16^+^ EVs in NAFLD patients with advanced fibrosis (F3-4) as compared to those with non-severe (F0-2) fibrosis, and the potential of these novel circulating markers to increase the diagnostic utility of the liver fibrosis score (LFS) (142). Although no NAFLD-patients were included, it is worth mentioning the study performed by Rautou et al, which identified significantly changing circulating levels of leuko-endothelial (CD31^+^/41^-^)-, pan-leukocyte (CD11a^+^)- and lymphocyte (CD4^+^)-derived EVs in patients with liver cirrhosis as compared to healthy controls. The number of leuko-endothelial (CD31^+^/41^-^)-derived EVs was found to be an indicator for systemic inflammation and the severity of cirrhosis (143). However, it is important to mention that the standardized preparation of EVs from plasma, their qualitative assessment (i.e., specific cargo content) as well as their accurate and reproducible quantification remain challenging and hamper the widespread use of EVs as NAFLD biomarkers at present (144).

Upon liver disease initiation and development, not only changing amounts of circulating EV subtypes and dynamic EV phenotypes can be observed, also significant changes in their miRNA cargo has been suggested as a potential diagnostic tool. While most studies focus on the total circulating miRNA content, consisting of protein (Ago2)-bound and EV-associated miRNAs, this diagnostic approach may lack sensitivity and specificity as delicate cell-type specific miRNA-dynamics may not be observed. Focusing on EV-associated miRNAs, representing the miRNA content of their cell of origin, may therefore be a more suitable diagnostic tool, as has been previously reported in a retrospective study using early-stage HBV/HCV patients (145). Indeed, various *in vitro* studies, mimicking the cellular changes observed during liver disease, identified a strong correlation between the dynamic cellular miRNA content, and the miRNA levels in their derived EVs (146–148). However, most of such studies have been executed using (primary) hepatocytes, and thus lack information concerning inflammatory cells and their EVs. Overall, upon the initiation and progression of liver disease, various circulating EV-associated miRNAs have dynamic expression levels (149). For example, EV-associated miRNA-122 has been found to increase in human NAFLD patients with significant liver fibrosis (150) and in dietary animal NASH models (151). Although most miRNAs are expressed by a variety of cell types, and show dynamics in various physiological or pathological conditions, some miRNAs may especially represent the increased inflammation associated with NAFLD-development. One such miRNA concerns miRNA-155, which is especially expressed in hepatocytes and macrophages, and which is known to control the innate and adaptive immune system during NAFLD (152). Indeed, miRNA-155 tightly contributes to TNFα production and LPS sensitization (152–154). Although its EV-associated expression levels have not yet been evaluated in human patients, increased levels are observed in LPS and CpG-administered mouse models of liver disease. Such enriched EV-associated miRNA-155 levels correlated well with the increased TNFα-levels and to the hepatic miRNA-155 content (155).

### Dynamic Changes in PBMC-Subsets

During the initiation and perpetuation of NAFLD, the important dynamic changes in the phenotype and quantity of circulating immune cells have been proposed as a potential non-invasive diagnostic tool. While some studies have tried to characterize the complete circulating white blood cell content (156), others mainly concentrate their research on peripheral blood mononuclear cells (PBMCs), consisting of lymphocytes and monocytes, as they have been found to display the most important dynamics during the NAFLD pathology. Indeed, PBMC-analysis of the peripheral blood of NAFLD patients compared to healthy individuals, identified a significant elevation in the total monocyte fraction (157). Moreover, quantitation of the three different monocyte subtypes, being the classical CD14^++^CD16^-^, intermediate CD14^++^CD16^+^ and non-classical CD14^+^CD16^++^ monocytes, identified an elevated number of non-classical monocytes, and a decreased number of classical monocytes in NAFLD patients. However, neither the quantitation of total monocyte levels, nor of monocyte subsets, was correlated to the severity of NASH (157–159). The different monocyte fractions showed strong association with age, triglyceride-content, and waist circumference (159). Interestingly, comparing monocyte-subsets between HCV- and NAFLD-patients, identified an increased proportion of non-classical monocytes only in the latter pathology (158).

Besides the important changes in monocyte subsets, a change in receptor/ligand expression on their cell membrane may be observed. For example, the expression of CCR4, which is known to recognise the chemoattractants CCL2, CCL4, and CCL5, is significantly enriched on monocytes, especially intermediate monocytes, from NAFLD patients as compared to healthy controls. Also sialic acid binding Ig-like lectin (SIGLEC)-1, also known as CD169, which is involved in monocyte recruitment, knows enrichment on intermediate and classical monocytes of NAFLD patients (158). Monocytes isolated from patients with NAFLD demonstrated increased expression of the inflammatory cytokines IL-6, TNF-a, and IL-1b, particularly in patients with fibrosis (160). Last, TLR6 expression is up-regulated on monocytes, and is useful as marker to differentiate NAFLD patients form obese individuals, and even to distinguish patients with NASH from those with simple steatosis (161).

Further characterisation of the circulating immune cell landscape led to the identification of decreased levels of total CD3^+^ cells, CD8^+^ T cells, CD56^dim^ NK cells, NKG2D^+^ NK cells, NKG2D^+^ CD56^dim^ NK cells, NKG2D^+^ iNKT cells, PD1^+^CD4^+^ T cells, CXCR3^+^CD4^+^ T cells, PD1^+^CD25^+^CD45RA^+^CD4^+^ T-cells, and mucosal-associated invariant T (MAIT) cells, and elevated numbers of total CD4^+^ T cells, CD25^+^CD45RA^+^CD4^+^ T-cells and Th2 cells, when NAFLD patients were compared to healthy controls (162, 163). Changing levels of PD1^+^CD4^+^, PD1^+^CD25^+^CD45RA^+^CD4^+^, and CXCR3^+^CD4^+^, CD25^+^CD45RA^+^CD4^+^ T-cells were even able to discriminate patients with significant fibrosis (F2-4) from those with no or mild fibrosis (163). Lastly, based on the observation of a diminished amount of resting Tregs (rTregs; CD4^+^CD45RA^+^CD25^++^) in patients with NASH, compared to those with NAFL, the Th17/rTreg and Th2/rTreg ratios have showed significant diagnostic value in NASH patients (164).

Overall, NAFLD affects the composition and functional properties of circulating immune cells, which likely reflects the hepatic pathology as well as extrahepatic metabolic disorders (e.g., in adipose tissue). While this is interesting from a conceptual perspective for understanding NASH, this has as of now not yet translated into a more accurate disease phenotyping or risk stratification. Part of this gap might be explained by technical challenges (e.g., requirement of fresh cells, laborious work-flow for multi-panel characterization), but part might be related to the complex biology, in which systemic immune responses are not solely driven by changes in the diseased liver.

## Concluding Remarks

One major hiatus in the clinical management of NAFLD patients remains the inability of non-invasive scoring tools to identify those patients with risk of NAFLD progression, and their lack in sensitivity and specificity to detect small changes in disease perpetuation or progression. An overwhelming number of studies have identified novel circulating markers, suggesting their utility for routine clinical practice. Indeed, in the search for such novel diagnostic tools, preference has been given to serological markers, as they harbor multiple features of the ideal biomarker, being their ease of sampling, wide availability, small sampling error, good cost-effectiveness, the possibility to execute repeated measures, possibility of automatization, and limited observer-related variability (11). Due to the important contribution of the inflammatory system in the NAFLD pathogenesis, circulating markers reflecting such inflammatory actions may represent innovative diagnostic tools.

One of the major drawbacks for the implementation of novel discovered biomarkers in clinical practice is their discovery in, often, small cross-sectional studies, lacking external validation. Very few studies investigated dynamic changes of inflammatory biomarkers in response to treatment, but the high number of ongoing clinical trials in NAFLD/NASH is expected to close this gap (165). Indeed, while the ideal patient cohort is heterogeneous in various parameters such as age, gender, and ethnicity, most of the reported studies rely on highly selected and specific patient populations, such as morbidly obese individuals. For example, while most studies have been performed in ethnicity-homogeneous populations, often with an Asian or Caucasian background, important ethnicity-dependent differences in the diagnostic utility of biomarkers have been reported (166). It is also important to mention that, due to the important perpetuating effects of type 2 diabetes mellitus (T2DM) on the risk of NAFLD and advanced fibrosis, the diagnostic utility of biomarkers may be altered due to presence of T2DM. Indeed, several studies reported that diagnostic tools, which were developed and validated in non-diabetic populations, may underperform when applied to NAFLD patients suffering from T2DM (167, 168). When comparing the results obtained from different studies, it is often difficult to compare the diagnostic utility of the proposed markers, due their validation against different standards, often liver biopsy or ultrasonography. Additionally, without the use of liver biopsy as reference, the credibility of the proposed marker is often lower, especially due to the inability to report the correlation of the marker with the various stages of NAFLD propagation and its histological-associated changes. When comparing studies concerning the same diagnostic marker, comparison of the negative and positive predictive value is often complicated due to the use of different cut-off values.

Due to the complex interplay of the various damaging “hits” in the pathogenesis of NASH, including the dysfunction/inflammation of adipose tissue and an overall increased systemic inflammation (169), circulating inflammatory markers are unable to provide information solely on hepatic inflammation, but instead represent the overall (hepatic and extrahepatic) inflammatory status. Significant differences in the level of extrahepatic inflammation between NASH-patients, e.g., lower levels of adipose tissue inflammation observed in NAFLD patients with the PNPLA3 I148M genetic variant as compared to weight-matched NAFLD patients homozygous for the wild type allele (170), may therefore cause the observed discrepancies between various published studies.

To gain popularity in the clinical community, a proposed novel biomarker should provide significant additional and useful information, impossible to obtain through use of the clinical routine parameters or imaging systems. While most of the clinical serological scoring tools are unable to differentiate patients with NAFLD from those with simple steatosis, some of the described inflammatory markers claim this possibility **(** **Table 1** **)**. However, almost all studies lack extensive validation to ensure credibility of these obtained results. It should also be mentioned that inflammatory markers are not liver-specific, and therefore demand critical interpretation, as they can be strongly influenced by comorbidities (171). As each individual biomarker has his own strengths and weaknesses, the combination of several of such circulating markers, or their combination with demographic characteristics or imaging-based results in the creation of mathematical models, probably holds the highest potential in the search for the ideal non-invasive diagnostic tool.

## Author Contributions

Concept and design, drafting of the manuscript, and critical revision by JL and FT. All authors contributed to the article and approved the submitted version.

## Funding

JL is supported by the Federal Ministry of Education and Research (BMBF, ImmuneAvatar), and FT is supported by the German Research Foundation (DFG SFB/TRR 296, CRC1382, Ta434/3-1, Ta434/5-1).

## Conflict of Interest

FT’s lab has received research funding by Allergan, Bristol-Myers Squibb, Galapagos, Gilead and Inventiva. He consults for Allergan, Bayer, Boehringer Ingelheim, Galapagos, Galmed, Intercept, Inventiva, NGM bio, Novartis and Pfizer.

The remaining author declares that the research was conducted in the absence of any commercial or financial relationships that could be construed as a potential conflict of interest.

## References

[1] Ibrahim SH Hirsova P Gores GJ Non-alcoholic steatohepatitis pathogenesis: sublethal hepatocyte injury as a driver of liver inflammation. Gut (2018) 67:963–72. 10.1136/gutjnl-2017-315691 PMC5889737 29367207

[2] L. European Association for the Study of the, D European Association for the Study of, and O European Association for the Study of, EASL-EASD-EASO Clinical Practice Guidelines for the management of non-alcoholic fatty liver disease. J Hepatol (2016) 64:1388–402. 10.1016/j.jhep.2015.11.004 27062661

[3] Estes C Anstee QM Arias-Loste MT Bantel H Bellentani S Caballeria J Modeling NAFLD disease burden in China, France, Germany, Italy, Japan, Spain, United Kingdom, and United States for the period 2016-2030. J Hepatol (2018) 69:896–904. 10.1016/j.jhep.2018.05.036 29886156

[4] Lambrecht J van Grunsven LA Tacke F Current and emerging pharmacotherapeutic interventions for the treatment of liver fibrosis. Expert Opin Pharmacother (2020) 4:1–13. 10.1080/14656566.2020.1774553 32543284

[5] Angulo P Kleiner DE Dam-Larsen S Adams LA Bjornsson ES Charatcharoenwitthaya P Liver Fibrosis, but No Other Histologic Features, Is Associated With Long-term Outcomes of Patients With Nonalcoholic Fatty Liver Disease. Gastroenterology (2015) 149:389–97 e10. 10.1053/j.gastro.2015.04.043 PMC4516664 25935633

[6] Dulai PS Singh S Patel J Soni M Prokop LJ Younossi Z Increased risk of mortality by fibrosis stage in nonalcoholic fatty liver disease: Systematic review and meta-analysis. Hepatology (2017) 65:1557–65. 10.1002/hep.29085 PMC5397356 28130788

[7] Rinella ME Tacke F Sanyal AJ Anstee QM A. E. W. participants of the Report on the AASLD/EASL joint workshop on clinical trial endpoints in NAFLD. J Hepatol (2019) 71:823–33. 10.1016/j.jhep.2019.04.019 31300231

[8] Brunt EM Kleiner DE Wilson LA Sanyal AJ Neuschwander-Tetri BA Nonalcoholic Steatohepatitis Clinical Research N Improvements in Histologic Features and Diagnosis Associated With Improvement in Fibrosis in Nonalcoholic Steatohepatitis: Results From the Nonalcoholic Steatohepatitis Clinical Research Network Treatment Trials. Hepatology (2019) 70:522–31. 10.1002/hep.30418 PMC6570584 30549292

[9] Kleiner DE Brunt EM Wilson LA Behling C Guy C Contos M Association of Histologic Disease Activity With Progression of Nonalcoholic Fatty Liver Disease. JAMA Netw Open (2019) 2:e1912565. 10.1001/jamanetworkopen.2019.12565 31584681 PMC6784786

[10] Schulz M Tacke F Identifying High-Risk NASH Patients: What We Know so Far. Hepat Med (2020) 12:125–38. 10.2147/HMER.S265473 PMC7493213 32982495

[11] Lambrecht J Verhulst S Mannaerts I Reynaert H van Grunsven LA Prospects in non-invasive assessment of liver fibrosis: Liquid biopsy as the future gold standard? Biochim Biophys Acta Mol Basis Dis (2018) 1864:1024–36. 10.1016/j.bbadis.2018.01.009 29329986

[12] Davison BA Harrison SA Cotter G Alkhouri N Sanyal A Edwards C Suboptimal reliability of liver biopsy evaluation has implications for randomized clinical trials. J Hepatol (2020) 73:1322–32. 10.1016/j.jhep.2020.06.025 32610115

[13] Piazzolla VA Mangia A Noninvasive Diagnosis of NAFLD and NASH. Cells (2020) 9:1005. 10.3390/cells9041005 PMC7226476 32316690

[14] Angulo P Hui JM Marchesini G Bugianesi E George J Farrell GC The NAFLD fibrosis score: a noninvasive system that identifies liver fibrosis in patients with NAFLD. Hepatology (2007) 45:846–54. 10.1002/hep.21496 17393509

[15] Xiao G Zhu S Xiao X Yan L Yang J Wu G Comparison of laboratory tests, ultrasound, or magnetic resonance elastography to detect fibrosis in patients with nonalcoholic fatty liver disease: A meta-analysis. Hepatology (2017) 66:1486–501. 10.1002/hep.29302 28586172

[16] L. European Association for Study of H. Asociacion Latinoamericana para el Estudio del EASL-ALEH Clinical Practice Guidelines: Non-invasive tests for evaluation of liver disease severity and prognosis. J Hepatol (2015) 63:237–64. 10.1016/j.jhep.2015.04.006 25911335

[17] Tapper EB Loomba R Noninvasive imaging biomarker assessment of liver fibrosis by elastography in NAFLD. Nat Rev Gastroenterol Hepatol (2018) 15:274–82. 10.1038/nrgastro.2018.10 PMC7504909 29463906

[18] Petta S Wong VW Camma C Hiriart JB Wong GL Marra F Improved noninvasive prediction of liver fibrosis by liver stiffness measurement in patients with nonalcoholic fatty liver disease accounting for controlled attenuation parameter values. Hepatology (2017) 65:1145–55. 10.1002/hep.28843 27639088

[19] de Ledinghen V Vergniol J Capdepont M Chermak F Hiriart JB Cassinotto C Controlled attenuation parameter (CAP) for the diagnosis of steatosis: a prospective study of 5323 examinations. J Hepatol (2014) 60:1026–31. 10.1016/j.jhep.2013.12.018 24378529

[20] Newsome PN Sasso M Deeks JJ Paredes A Boursier J Chan WK FibroScan-AST (FAST) score for the non-invasive identification of patients with non-alcoholic steatohepatitis with significant activity and fibrosis: a prospective derivation and global validation study. Lancet Gastroenterol Hepatol (2020) 5:362–73. 10.1016/S2468-1253(19)30383-8 PMC7066580 32027858

[21] Imajo K Kessoku T Honda Y Tomeno W Ogawa Y Mawatari H Magnetic Resonance Imaging More Accurately Classifies Steatosis and Fibrosis in Patients With Nonalcoholic Fatty Liver Disease Than Transient Elastography. Gastroenterology (2016) 150:626–37 e7. 10.1053/j.gastro.2015.11.048 26677985

[22] Park CC Nguyen P Hernandez C Bettencourt R Ramirez K Fortney L Magnetic Resonance Elastography vs Transient Elastography in Detection of Fibrosis and Noninvasive Measurement of Steatosis in Patients With Biopsy-Proven Nonalcoholic Fatty Liver Disease. Gastroenterology (2017) 152:598–607.e2. 10.1053/j.gastro.2016.10.026 27911262 PMC5285304

[23] Angulo P Bugianesi E Bjornsson ES Charatcharoenwitthaya P Mills PR Barrera F Simple noninvasive systems predict long-term outcomes of patients with nonalcoholic fatty liver disease. Gastroenterology (2013) 145:782–9 e4. 10.1053/j.gastro.2013.06.057 23860502 PMC3931256

[24] Sanyal AJ Past, present and future perspectives in nonalcoholic fatty liver disease. Nat Rev Gastroenterol Hepatol (2019) 16:377–86. 10.1038/s41575-019-0144-8 31024089

[25] Cordeiro A Costa R Andrade N Silva C Canabrava N Pena MJ Does adipose tissue inflammation drive the development of non-alcoholic fatty liver disease in obesity? Clin Res Hepatol Gastroenterol (2020) 44:394–402. 10.1016/j.clinre.2019.10.001 32044284

[26] Gastaldelli A Cusi K From NASH to diabetes and from diabetes to NASH: Mechanisms and treatment options. JHEP Rep (2019) 1:312–28. 10.1016/j.jhepr.2019.07.002 PMC7001557 32039382

[27] du Plessis J van Pelt J Korf H Mathieu C van der Schueren B Lannoo M Association of Adipose Tissue Inflammation With Histologic Severity of Nonalcoholic Fatty Liver Disease. Gastroenterology (2015) 149:635–48 e14. 10.1053/j.gastro.2015.05.044 26028579

[28] Krenkel O Puengel T Govaere O Abdallah AT Mossanen JC Kohlhepp M Therapeutic inhibition of inflammatory monocyte recruitment reduces steatohepatitis and liver fibrosis. Hepatology (2018) 67:1270–83. 10.1002/hep.29544 28940700

[29] Lefere S Tacke F Macrophages in obesity and non-alcoholic fatty liver disease: Crosstalk with metabolism. JHEP Rep (2019) 1:30–43. 10.1016/j.jhepr.2019.02.004 32149275 PMC7052781

[30] Tilg H Adolph TE Moschen AR Multiple Parallel Hits Hypothesis in NAFLD - Revisited After a Decade. Hepatology (2020). 10.1002/hep.31518 PMC7898624 32780879

[31] Parola M Pinzani M Liver fibrosis: Pathophysiology, pathogenetic targets and clinical issues. Mol Aspects Med (2019) 65:37–55. 10.1016/j.mam.2018.09.002 30213667

[32] Wen Y Lambrecht J Ju C Tacke F Hepatic macrophages in liver homeostasis and diseases-diversity, plasticity and therapeutic opportunities. Cell Mol Immunol (2020) 82:45–6. 10.1038/s41423-020-00558-8 PMC7852525 33041338

[33] Lang S Schnabl B Microbiota and Fatty Liver Disease-the Known, the Unknown, and the Future. Cell Host Microbe (2020) 28:233–44. 10.1016/j.chom.2020.07.007 PMC7467841 32791115

[34] Lang S Demir M Martin A Jiang L Zhang X Duan Y Intestinal Virome Signature Associated With Severity of Nonalcoholic Fatty Liver Disease. Gastroenterology (2020) 159:1839–52. 10.1053/j.gastro.2020.07.005 PMC8404510 32652145

[35] Marra F Svegliati-Baroni G Lipotoxicity and the gut-liver axis in NASH pathogenesis. J Hepatol (2018) 68:280–95. 10.1016/j.jhep.2017.11.014 29154964

[36] Itoh M Suganami T Kato H Kanai S Shirakawa I Sakai T CD11c+ resident macrophages drive hepatocyte death-triggered liver fibrosis in a murine model of nonalcoholic steatohepatitis. JCI Insight (2017) 2:e92902. 10.1172/jci.insight.92902 PMC5752377 29202448

[37] Miura K Yang L van Rooijen N Ohnishi H Seki E Hepatic recruitment of macrophages promotes nonalcoholic steatohepatitis through CCR2. Am J Physiol Gastrointest Liver Physiol (2012) 302:G1310–21. 10.1152/ajpgi.00365.2011 PMC3378163 22442158

[38] Hossain M Kubes P Innate immune cells orchestrate the repair of sterile injury in the liver and beyond. Eur J Immunol (2019) 49:831–41. 10.1002/eji.201847485 31001813

[39] Koruk M Taysi S Savas MC Yilmaz O Akcay F Karakok M Serum levels of acute phase proteins in patients with nonalcoholic steatohepatitis. Turk J Gastroenterol (2003) 14:12–7. 14593532

[40] Yoneda M Mawatari H Fujita K Iida H Yonemitsu K Kato S High-sensitivity C-reactive protein is an independent clinical feature of nonalcoholic steatohepatitis (NASH) and also of the severity of fibrosis in NASH. J Gastroenterol (2007) 42:573–82. 10.1007/s00535-007-2060-x 17653654

[41] Riquelme A Arrese M Soza A Morales A Baudrand R Perez-Ayuso RM Non-alcoholic fatty liver disease and its association with obesity, insulin resistance and increased serum levels of C-reactive protein in Hispanics. Liver Int (2009) 29:82–8. 10.1111/j.1478-3231.2008.01823.x 18647235

[42] Park SH Kim BI Yun JW Kim JW Park DI Cho YK Insulin resistance and C-reactive protein as independent risk factors for non-alcoholic fatty liver disease in non-obese Asian men. J Gastroenterol Hepatol (2004) 19:694–8. 10.1111/j.1440-1746.2004.03362.x 15151626

[43] Chiang CH Huang CC Chan WL Chen JW Leu HB The severity of non-alcoholic fatty liver disease correlates with high sensitivity C-reactive protein value and is independently associated with increased cardiovascular risk in healthy population. Clin Biochem (2010) 43:1399–404. 10.1016/j.clinbiochem.2010.09.003 20846522

[44] Ridker PM Hennekens CH Buring JE Rifai N C-reactive protein and other markers of inflammation in the prediction of cardiovascular disease in women. N Engl J Med (2000) 342:836–43. 10.1056/NEJM200003233421202 10733371

[45] Hui JM Farrell GC Kench JG George J High sensitivity C-reactive protein values do not reliably predict the severity of histological changes in NAFLD. Hepatology (2004) 39:1458–9. 10.1002/hep.20223 15122781

[46] Zimmermann E Anty R Tordjman J Verrijken A Gual P Tran A C-reactive protein levels in relation to various features of non-alcoholic fatty liver disease among obese patients. J Hepatol (2011) 55:660–5. 10.1016/j.jhep.2010.12.017 21238518

[47] Haukeland JW Damas JK Konopski Z Loberg EM Haaland T Goverud I Systemic inflammation in nonalcoholic fatty liver disease is characterized by elevated levels of CCL2. J Hepatol (2006) 44:1167–74. 10.1016/j.jhep.2006.02.011 16618517

[48] Targher G Bertolini L Scala L Zoppini G Zenari L Falezza G Non-alcoholic hepatic steatosis and its relation to increased plasma biomarkers of inflammation and endothelial dysfunction in non-diabetic men. Role of visceral adipose tissue. Diabetes Med (2005) 22:1354–8. 10.1111/j.1464-5491.2005.01646.x 16176196

[49] Timpson NJ Lawlor DA Harbord RM Gaunt TR Day IN Palmer LJ C-reactive protein and its role in metabolic syndrome: mendelian randomisation study. Lancet (2005) 366:1954–9. 10.1016/S0140-6736(05)67786-0 16325697

[50] Pearce SG Thosani NC Pan JJ Noninvasive biomarkers for the diagnosis of steatohepatitis and advanced fibrosis in NAFLD. Biomark Res (2013) 1:7. 10.1186/2050-7771-1-7 24252302 PMC4177607

[51] Boga S Koksal AR Alkim H Yilmaz Ozguven MB Bayram M Ergun M Plasma Pentraxin 3 Differentiates Nonalcoholic Steatohepatitis (NASH) from Non-NASH. Metab Syndr Relat Disord (2015) 13:393–9. 10.1089/met.2015.0046 26367098

[52] Gurel H Genc H Celebi G Sertoglu E Cicek AF Kayadibi H Plasma pentraxin-3 is associated with endothelial dysfunction in non-alcoholic fatty liver disease. Eur Rev Med Pharmacol Sci (2016) 20:4305–12. 27831642

[53] Trojak A Walus-Miarka M Kapusta M Miarka P Kawalec E Idzior-Walus B Serum pentraxin 3 concentration in patients with type 2 diabetes and nonalcoholic fatty liver disease. Pol Arch Intern Med (2019) 129:499–505. 10.20452/pamw.14913 31469122

[54] Yoneda M Uchiyama T Kato S Endo H Fujita K Yoneda K Plasma Pentraxin3 is a novel marker for nonalcoholic steatohepatitis (NASH). BMC Gastroenterol (2008) 8:53. 10.1186/1471-230X-8-53 19014569 PMC2621235

[55] Ozturk K Kurt O Dogan T Ozen A Demirci H Yesildal F Pentraxin 3 Is a Predictor for Fibrosis and Arterial Stiffness in Patients with Nonalcoholic Fatty Liver Disease. Gastroenterol Res Pract (2016) 2016:1417962. 10.1155/2016/1417962 26997950 PMC4779836

[56] Hamza RT Elfaramawy AA Mahmoud NH Serum Pentraxin 3 Fragment as a Noninvasive Marker of Nonalcoholic Fatty Liver Disease in Obese Children and Adolescents. Horm Res Paediatr (2016) 86:11–20. 10.1159/000446566 27309736

[57] Maleki I Rastgar A Hosseini V Taghvaei T Rafiei A Barzin M High sensitive CRP and pentraxine 3 as noninvasive biomarkers of nonalcoholic fatty liver disease. Eur Rev Med Pharmacol Sci (2014) 18:1583–90. 24943967

[58] Katsarou A Moustakas II Pyrina I Lembessis P Koutsilieris M Chatzigeorgiou A Metabolic inflammation as an instigator of fibrosis during non-alcoholic fatty liver disease. World J Gastroenterol (2020) 26:1993–2011. 10.3748/wjg.v26.i17.1993 32536770 PMC7267690

[59] Mirea AM Tack CJ Chavakis T Joosten LAB Toonen EJM IL-1 Family Cytokine Pathways Underlying NAFLD: Towards New Treatment Strategies. Trends Mol Med (2018) 24:458–71. 10.1016/j.molmed.2018.03.005 PMC5939989 29665983

[60] Paquissi FC Immune Imbalances in Non-Alcoholic Fatty Liver Disease: From General Biomarkers and Neutrophils to Interleukin-17 Axis Activation and New Therapeutic Targets. Front Immunol (2016) 7:490. 10.3389/fimmu.2016.00490 27891128 PMC5104753

[61] Jarrar MH Baranova A Collantes R Ranard B Stepanova M Bennett C Adipokines and cytokines in non-alcoholic fatty liver disease. Aliment Pharmacol Ther (2008) 27:412–21. 10.1111/j.1365-2036.2007.03586.x 18081738

[62] Fricker ZP Pedley A Massaro JM Vasan RS Hoffmann U Benjamin EJ Liver Fat Is Associated With Markers of Inflammation and Oxidative Stress in Analysis of Data From the Framingham Heart Study. Clin Gastroenterol Hepatol (2019) 17:57–64.e4. 10.1016/j.cgh.2018.11.037 PMC6475462 30476583

[63] Abiru S Migita K Maeda Y Daikoku M Ito M Ohata K Serum cytokine and soluble cytokine receptor levels in patients with non-alcoholic steatohepatitis. Liver Int (2006) 26:39–45. 10.1111/j.1478-3231.2005.01191.x 16420507

[64] Kar S Paglialunga S Jaycox SH Islam R Paredes AH Assay validation and clinical performance of chronic inflammatory and chemokine biomarkers of NASH fibrosis. PloS One (2019) 14:e0217263. 10.1371/journal.pone.0217263 31291245 PMC6619600

[65] Wieckowska A Papouchado BG Li Z Lopez R Zein NN Feldstein AE Increased hepatic and circulating interleukin-6 levels in human nonalcoholic steatohepatitis. Am J Gastroenterol (2008) 103:1372–9. 10.1111/j.1572-0241.2007.01774.x 18510618

[66] Zimmermann HW Seidler S Gassler N Nattermann J Luedde T Trautwein C Interleukin-8 is activated in patients with chronic liver diseases and associated with hepatic macrophage accumulation in human liver fibrosis. PloS One (2011) 6:e21381. 10.1371/journal.pone.0021381 21731723 PMC3120868

[67] Glass O Henao R Patel K Guy CD Gruss HJ Syn WK Serum Interleukin-8, Osteopontin, and Monocyte Chemoattractant Protein 1 Are Associated With Hepatic Fibrosis in Patients With Nonalcoholic Fatty Liver Disease. Hepatol Commun (2018) 2:1344–55. 10.1002/hep4.1237 PMC6211321 30411081

[68] Ajmera V Perito ER Bass NM Terrault NA Yates KP Gill R Novel plasma biomarkers associated with liver disease severity in adults with nonalcoholic fatty liver disease. Hepatology (2017) 65:65–77. 10.1002/hep.28776 27532276 PMC5191932

[69] Auguet T Bertran L Binetti J Aguilar C Martinez S Sabench F Relationship between IL-8 Circulating Levels and TLR2 Hepatic Expression in Women with Morbid Obesity and Nonalcoholic Steatohepatitis. Int J Mol Sci (2020) 21:4189. 10.3390/ijms21114189 PMC7312372 32545403

[70] Hammerich L Tacke F Interleukins in chronic liver disease: lessons learned from experimental mouse models. Clin Exp Gastroenterol (2014) 7:297–306. 10.2147/CEG.S43737 25214799 PMC4158890

[71] Darmadi D Ruslie RH Association between serum interleukin (IL)-12 level and severity of non-alcoholic fatty liver disease (NAFLD). Rom J Intern Med (2020). 10.2478/rjim-2020-0029 33055315

[72] Dali-Youcef N Vix M Costantino F El-Saghire H Lhermitte B Callari C Interleukin-32 Contributes to Human Nonalcoholic Fatty Liver Disease and Insulin Resistance. Hepatol Commun (2019) 3:1205–20. 10.1002/hep4.1396 PMC6719754 31497742

[73] Baselli GA Dongiovanni P Rametta R Meroni M Pelusi S Maggioni M Liver transcriptomics highlights interleukin-32 as novel NAFLD-related cytokine and candidate biomarker. Gut (2020) 69:1855–66. 10.1136/gutjnl-2019-319226 PMC7497582 32001554

[74] Knorr J Wree A Tacke F Feldstein AE The NLRP3 Inflammasome in Alcoholic and Nonalcoholic Steatohepatitis. Semin Liver Dis (2020) 40:298–306. 10.1055/s-0040-1708540 32526788

[75] Hadinia A Doustimotlagh AH Goodarzi HR Arya A Jafarinia M Circulating Levels of Pro-inflammatory Cytokines in Patients with Nonalcoholic Fatty Liver Disease and Non-Alcoholic Steatohepatitis. Iran J Immunol (2019) 16:327–33. 10.22034/IJI.2019.80284 31885010

[76] He K Zhu X Liu Y Miao C Wang T Li P Inhibition of NLRP3 inflammasome by thioredoxin-interacting protein in mouse Kupffer cells as a regulatory mechanism for non-alcoholic fatty liver disease development. Oncotarget (2017) 8:37657–72. 10.18632/oncotarget.17489 PMC5514938 28499273

[77] Pihlajamaki J Kuulasmaa T Kaminska D Simonen M Karja V Gronlund S Serum interleukin 1 receptor antagonist as an independent marker of non-alcoholic steatohepatitis in humans. J Hepatol (2012) 56:663–70. 10.1016/j.jhep.2011.10.005 22027586

[78] Hung J McQuillan BM Chapman CM Thompson PL Beilby JP Elevated interleukin-18 levels are associated with the metabolic syndrome independent of obesity and insulin resistance. Arterioscler Thromb Vasc Biol (2005) 25:1268–73. 10.1161/01.ATV.0000163843.70369.12 15790931

[79] Vecchiet J Falasca K Cacciatore P Zingariello P Dalessandro M Marinopiccoli M Association between plasma interleukin-18 levels and liver injury in chronic hepatitis C virus infection and non-alcoholic fatty liver disease. Ann Clin Lab Sci (2005) 35:415–22. 16254258

[80] Tapan S Dogru T Kara M Ercin CN Kilciler G Genc H Circulating levels of interleukin-18 in patients with non-alcoholic fatty liver disease. Scand J Clin Lab Invest (2010) 70:399–403. 10.3109/00365513.2010.500675 20604719

[81] Hohenester S Kanitz V Schiergens T Einer C Nagel J Wimmer R IL-18 but Not IL-1 Signaling Is Pivotal for the Initiation of Liver Injury in Murine Non-Alcoholic Fatty Liver Disease. Int J Mol Sci (2020) 21:8602. 10.3390/ijms21228602 PMC7696705 33202693

[82] Galli SJ Borregaard N Wynn TA Phenotypic and functional plasticity of cells of innate immunity: macrophages, mast cells and neutrophils. Nat Immunol (2011) 12:1035–44. 10.1038/ni.2109 PMC3412172 22012443

[83] Yilmaz H Yalcin KS Namuslu M Celik HT Sozen M Inan O Neutrophil-Lymphocyte Ratio (NLR) Could Be Better Predictor than C-reactive Protein (CRP) for Liver Fibrosis in Non-alcoholic Steatohepatitis(NASH). Ann Clin Lab Sci (2015) 45:278–86. 26116591

[84] Abdel-Razik A Mousa N Shabana W Refaey M ElMahdy Y Elhelaly R A novel model using mean platelet volume and neutrophil to lymphocyte ratio as a marker of nonalcoholic steatohepatitis in NAFLD patients: multicentric study. Eur J Gastroenterol Hepatol (2016) 28:e1–9. 10.1097/MEG.0000000000000486 26469357

[85] Alkhouri N Morris-Stiff G Campbell C Lopez R Tamimi TA Yerian L Neutrophil to lymphocyte ratio: a new marker for predicting steatohepatitis and fibrosis in patients with nonalcoholic fatty liver disease. Liver Int (2012) 32:297–302. 10.1111/j.1478-3231.2011.02639.x 22097893

[86] Wu L Gao X Guo Q Li J Yao J Yan K The role of neutrophils in innate immunity-driven nonalcoholic steatohepatitis: lessons learned and future promise. Hepatol Int (2020) 14:652–66. 10.1007/s12072-020-10081-7 32880077

[87] Leithead JA Rajoriya N Gunson BK Ferguson JW Neutrophil-to-lymphocyte ratio predicts mortality in patients listed for liver transplantation. Liver Int (2015) 35:502–9. 10.1111/liv.12688 25234369

[88] Wang Y Small lipid-binding proteins in regulating endothelial and vascular functions: focusing on adipocyte fatty acid binding protein and lipocalin-2. Br J Pharmacol (2012) 165:603–21. 10.1111/j.1476-5381.2011.01528.x PMC3315034 21658023

[89] Ye Z Wang S Yang Z He M Zhang S Zhang W Serum lipocalin-2, cathepsin S and chemerin levels and nonalcoholic fatty liver disease. Mol Biol Rep (2014) 41:1317–23. 10.1007/s11033-013-2977-5 24390241

[90] Ye D Yang K Zang S Lin Z Chau HT Wang Y Lipocalin-2 mediates non-alcoholic steatohepatitis by promoting neutrophil-macrophage crosstalk via the induction of CXCR2. J Hepatol (2016) 65:988–97. 10.1016/j.jhep.2016.05.041 27266617

[91] Xu MJ Feng D Wu H Wang H Chan Y Kolls J Liver is the major source of elevated serum lipocalin-2 levels after bacterial infection or partial hepatectomy: a critical role for IL-6/STAT3. Hepatology (2015) 61:692–702. 10.1002/hep.27447 25234944 PMC4303493

[92] Xiao X Yeoh BS Vijay-Kumar M Lipocalin 2: An Emerging Player in Iron Homeostasis and Inflammation. Annu Rev Nutr (2017) 37:103–30. 10.1146/annurev-nutr-071816-064559 28628361

[93] Syn WK Choi SS Diehl AM Apoptosis and cytokines in non-alcoholic steatohepatitis. Clin Liver Dis (2009) 13:565–80. 10.1016/j.cld.2009.07.003 PMC2766093 19818305

[94] Koca SS Bahcecioglu IH Poyrazoglu OK Ozercan IH Sahin K Ustundag B The treatment with antibody of TNF-alpha reduces the inflammation, necrosis and fibrosis in the non-alcoholic steatohepatitis induced by methionine- and choline-deficient diet. Inflammation (2008) 31:91–8. 10.1007/s10753-007-9053-z 18066656

[95] Barbuio R Milanski M Bertolo MB Saad MJ Velloso LA Infliximab reverses steatosis and improves insulin signal transduction in liver of rats fed a high-fat diet. J Endocrinol (2007) 194:539–50. 10.1677/JOE-07-0234 17761893

[96] Wandrer F Liebig S Marhenke S Vogel A John K Manns MP TNF-Receptor-1 inhibition reduces liver steatosis, hepatocellular injury and fibrosis in NAFLD mice. Cell Death Dis (2020) 11:212. 10.1038/s41419-020-2411-6 32235829 PMC7109108

[97] Daijo K Nakahara T Inagaki Y Nanba M Nishida Y Uchikawa S Risk factors for histological progression of non-alcoholic steatohepatitis analyzed from repeated biopsy cases. J Gastroenterol Hepatol (2020) 35:1412–9. 10.1111/jgh.14968 31896166

[98] Ajmal MR Yaccha M Malik MA Rabbani MU Ahmad I Isalm N Prevalence of nonalcoholic fatty liver disease (NAFLD) in patients of cardiovascular diseases and its association with hs-CRP and TNF-alpha. Indian Heart J (2014) 66:574–9. 10.1016/j.ihj.2014.08.006 PMC4310973 25634387

[99] Alaaeddine N Sidaoui J Hilal G Serhal R Abedelrahman A Khoury S TNF-alpha messenger ribonucleic acid (mRNA) in patients with nonalcoholic steatohepatitis. Eur Cytokine Netw (2012) 23:107–11. 10.1684/ecn.2012.0313 23009757

[100] Bahcecioglu IH Yalniz M Ataseven H Ilhan N Ozercan IH Seckin D Levels of serum hyaluronic acid, TNF-alpha and IL-8 in patients with nonalcoholic steatohepatitis. Hepatogastroenterology (2005) 52:1549–53. 16201116

[101] Tokushige K Takakura M Tsuchiya-Matsushita N Taniai M Hashimoto E Shiratori K Influence of TNF gene polymorphisms in Japanese patients with NASH and simple steatosis. J Hepatol (2007) 46:1104–10. 10.1016/j.jhep.2007.01.028 17395331

[102] Tokushige K Hashimoto E Tsuchiya N Kaneda H Taniai M Shiratori K Clinical significance of soluble TNF receptor in Japanese patients with non-alcoholic steatohepatitis. Alcohol Clin Exp Res (2005) 29:298S–303S. 10.1097/01.alc.0000191810.46000.37 16385240

[103] Lebensztejn DM Kowalczuk D Tarasow E Skiba E Kaczmarski M Tumor necrosis factor alpha and its soluble receptors in obese children with NAFLD. Adv Med Sci (2010) 55:74–9. 10.2478/v10039-010-0008-5 20371430

[104] Manco M Marcellini M Giannone G Nobili V Correlation of serum TNF-alpha levels and histologic liver injury scores in pediatric nonalcoholic fatty liver disease. Am J Clin Pathol (2007) 127:954–60. 10.1309/6VJ4DWGYDU0XYJ8Q 17509993

[105] Zimmermann HW Seidler S Nattermann J Gassler N Hellerbrand C Zernecke A Functional contribution of elevated circulating and hepatic non-classical CD14CD16 monocytes to inflammation and human liver fibrosis. PloS One (2010) 5:e11049. 10.1371/journal.pone.0011049 20548789 PMC2883575

[106] Wright SD Ramos RA Tobias PS Ulevitch RJ Mathison JC CD14, a receptor for complexes of lipopolysaccharide (LPS) and LPS binding protein. Science (1990) 249:1431–3. 10.1126/science.1698311 1698311

[107] Pugin J Heumann ID Tomasz A Kravchenko VV Akamatsu Y Nishijima M CD14 is a pattern recognition receptor. Immunity (1994) 1:509–16. 10.1016/1074-7613(94)90093-0 7534618

[108] Tonan T Fujimoto K Qayyum A Morita Y Nakashima O Ono N CD14 expression and Kupffer cell dysfunction in non-alcoholic steatohepatitis: superparamagnetic iron oxide-magnetic resonance image and pathologic correlation. J Gastroenterol Hepatol (2012) 27:789–96. 10.1111/j.1440-1746.2011.07057.x 22188204

[109] Kapil S Duseja A Sharma BK Singla B Chakraborti A Das A Genetic polymorphism in CD14 gene, a co-receptor of TLR4 associated with non-alcoholic fatty liver disease. World J Gastroenterol (2016) 22:9346–55. 10.3748/wjg.v22.i42.9346 PMC5107698 27895422

[110] Pan Z Zhou L Hetherington CJ Zhang DE Hepatocytes contribute to soluble CD14 production, and CD14 expression is differentially regulated in hepatocytes and monocytes. J Biol Chem (2000) 275:36430–5. 10.1074/jbc.M003192200 10960472

[111] Fernandez-Real JM Lopez-Bermejo A Broch M Vendrell J Richart C Ricart W Circulating soluble CD14 monocyte receptor is associated with increased alanine aminotransferase. Clin Chem (2004) 50:1456–8. 10.1373/clinchem.2003.030015 15277359

[112] Fernandez-Real JM Broch M Richart C Vendrell J Lopez-Bermejo A Ricart W CD14 monocyte receptor, involved in the inflammatory cascade, and insulin sensitivity. J Clin Endocrinol Metab (2003) 88:1780–4. 10.1210/jc.2002-020173 12679473

[113] Manco M Fernandez-Real JM Vecchio FM Vellone V Moreno JM Tondolo V The decrease of serum levels of human neutrophil alpha-defensins parallels with the surgery-induced amelioration of NASH in obesity. Obes Surg (2010) 20:1682–9. 10.1007/s11695-010-0129-8 20379797

[114] Ogawa Y Imajo K Yoneda M Kessoku T Tomeno W Shinohara Y Soluble CD14 levels reflect liver inflammation in patients with nonalcoholic steatohepatitis. PloS One (2013) 8:e65211. 10.1371/journal.pone.0065211 23762319 PMC3676404

[115] du Plessis J Korf H van Pelt J Windmolders P Vander Elst I Verrijken A Pro-Inflammatory Cytokines but Not Endotoxin-Related Parameters Associate with Disease Severity in Patients with NAFLD. PloS One (2016) 11:e0166048. 10.1371/journal.pone.0166048 27992443 PMC5167229

[116] Silverstein RL Febbraio M CD36, a scavenger receptor involved in immunity, metabolism, angiogenesis, and behavior. Sci Signal (2009) 2:re3. 10.1126/scisignal.272re3 19471024 PMC2811062

[117] Rada P Gonzalez-Rodriguez A Garcia-Monzon C Valverde AM Understanding lipotoxicity in NAFLD pathogenesis: is CD36 a key driver? Cell Death Dis (2020) 11:802. 10.1038/s41419-020-03003-w 32978374 PMC7519685

[118] He J Lee JH Febbraio M Xie W The emerging roles of fatty acid translocase/CD36 and the aryl hydrocarbon receptor in fatty liver disease. Exp Biol Med (Maywood) (2011) 236:1116–21. 10.1258/ebm.2011.011128 21885479

[119] Greco D Kotronen A Westerbacka J Puig O Arkkila P Kiviluoto T Gene expression in human NAFLD. Am J Physiol Gastrointest Liver Physiol (2008) 294:G1281–7. 10.1152/ajpgi.00074.2008 18388185

[120] Bechmann LP Gieseler RK Sowa JP Kahraman A Erhard J Wedemeyer I Apoptosis is associated with CD36/fatty acid translocase upregulation in non-alcoholic steatohepatitis. Liver Int (2010) 30:850–9. 10.1111/j.1478-3231.2010.02248.x 20408954

[121] Miquilena-Colina ME Lima-Cabello E Sanchez-Campos S Garcia-Mediavilla MV Fernandez-Bermejo M Lozano-Rodriguez T Hepatic fatty acid translocase CD36 upregulation is associated with insulin resistance, hyperinsulinaemia and increased steatosis in non-alcoholic steatohepatitis and chronic hepatitis C. Gut (2011) 60:1394–402. 10.1136/gut.2010.222844 21270117

[122] Sheedfar F Sung MM Aparicio-Vergara M Kloosterhuis NJ Miquilena-Colina ME Vargas-Castrillon J Increased hepatic CD36 expression with age is associated with enhanced susceptibility to nonalcoholic fatty liver disease. Aging (Albany NY) (2014) 6:281–95. 10.18632/aging.100652 PMC4032795 24751397

[123] Garcia-Monzon C Lo Iacono O Crespo J Romero-Gomez M Garcia-Samaniego J Fernandez-Bermejo M Increased soluble CD36 is linked to advanced steatosis in nonalcoholic fatty liver disease. Eur J Clin Invest (2014) 44:65–73. 10.1111/eci.12192 24134687

[124] Handberg A Hojlund K Gastaldelli A Flyvbjerg A Dekker JM Petrie J Plasma sCD36 is associated with markers of atherosclerosis, insulin resistance and fatty liver in a nondiabetic healthy population. J Intern Med (2012) 271:294–304. 10.1111/j.1365-2796.2011.02442.x 21883535

[125] Glintborg D Hojlund K Andersen M Henriksen JE Beck-Nielsen H Handberg A Soluble CD36 and risk markers of insulin resistance and atherosclerosis are elevated in polycystic ovary syndrome and significantly reduced during pioglitazone treatment. Diabetes Care (2008) 31:328–34. 10.2337/dc07-1424 18000176

[126] Handberg A Levin K Hojlund K Beck-Nielsen H Identification of the oxidized low-density lipoprotein scavenger receptor CD36 in plasma: a novel marker of insulin resistance. Circulation (2006) 114:1169–76. 10.1161/CIRCULATIONAHA.106.626135 16952981

[127] Nielsen MC Hvidbjerg Gantzel R Claria J Trebicka J Moller HJ Gronbaek H Macrophage Activation Markers, CD163 and CD206, in Acute-on-Chronic Liver Failure. Cells (2020) 9:1175. 10.3390/cells9051175 PMC7290463 32397365

[128] Moller HJ Soluble CD163. Scand J Clin Lab Invest (2012) 72:1–13. 10.3109/00365513.2011.626868 22060747

[129] Hintz KA Rassias AJ Wardwell K Moss ML Morganelli PM Pioli PA Endotoxin induces rapid metalloproteinase-mediated shedding followed by up-regulation of the monocyte hemoglobin scavenger receptor CD163. J Leukoc Biol (2002) 72:711–7. 10.1189/jlb.72.4.711 12377940

[130] Kazankov K Tordjman J Moller HJ Vilstrup H Poitou C Bedossa P Macrophage activation marker soluble CD163 and non-alcoholic fatty liver disease in morbidly obese patients undergoing bariatric surgery. J Gastroenterol Hepatol (2015) 30:1293–300. 10.1111/jgh.12943 25772748

[131] Bauer S Weiss TS Wiest R Schacherer D Hellerbrand C Farkas S Soluble CD163 is not increased in visceral fat and steatotic liver and is even suppressed by free fatty acids in vitro. Exp Mol Pathol (2011) 91:733–9. 10.1016/j.yexmp.2011.07.005 21839737

[132] Hegazy MA Mogawer SM Alnaggar A Ghoniem OA Abdel Samie RM Serum LPS and CD163 Biomarkers Confirming the Role of Gut Dysbiosis in Overweight Patients with NASH. Diabetes Metab Syndr Obes (2020) 13:3861–72. 10.2147/DMSO.S249949 PMC7585799 33116732

[133] Mueller JL Feeney ER Zheng H Misdraji J Kruger AJ Alatrakchi N Circulating Soluble CD163 is Associated with Steatohepatitis and Advanced Fibrosis in Nonalcoholic Fatty Liver Disease. Clin Transl Gastroenterol (2015) 6:e114. 10.1038/ctg.2015.36 26448455 PMC4816035

[134] Kazankov K Barrera F Moller HJ Rosso C Bugianesi E David E The macrophage activation marker sCD163 is associated with morphological disease stages in patients with non-alcoholic fatty liver disease. Liver Int (2016) 36:1549–57. 10.1111/liv.13150 27102725

[135] Rodgaard-Hansen S St George A Kazankov K Bauman A George J Gronbaek H Effects of lifestyle intervention on soluble CD163, a macrophage activation marker, in patients with non-alcoholic fatty liver disease. Scand J Clin Lab Invest (2017) 77:498–504. 10.1080/00365513.2017.1346823 28715286

[136] Fjeldborg K Christiansen T Bennetzen M H JM Pedersen SB Richelsen B The macrophage-specific serum marker, soluble CD163, is increased in obesity and reduced after dietary-induced weight loss. Obesity (Silver Spring) (2013) 21:2437–43. 10.1002/oby.20376 23512476

[137] De Vito R Alisi A Masotti A Ceccarelli S Panera N Citti A Markers of activated inflammatory cells correlate with severity of liver damage in children with nonalcoholic fatty liver disease. Int J Mol Med (2012) 30:49–56. 10.3892/ijmm.2012.965 22505182

[138] Kazankov K Moller HJ Lange A Birkebaek NH Holland-Fischer P Solvig J The macrophage activation marker sCD163 is associated with changes in NAFLD and metabolic profile during lifestyle intervention in obese children. Pediatr Obes (2015) 10:226–33. 10.1111/ijpo.252 25073966

[139] Povero D Yamashita H Ren W Subramanian MG Myers RP Eguchi A Characterization and Proteome of Circulating Extracellular Vesicles as Potential Biomarkers for NASH. Hepatol Commun (2020) 4:1263–78. 10.1002/hep4.1556 PMC7471415 32923831

[140] Li J Liu H Mauer AS Lucien F Raiter A Bandla H Characterization of Cellular Sources and Circulating Levels of Extracellular Vesicles in a Dietary Murine Model of Nonalcoholic Steatohepatitis. Hepatol Commun (2019) 3:1235–49. 10.1002/hep4.1404 PMC6719742 31497744

[141] Kornek M Lynch M Mehta SH Lai M Exley M Afdhal NH Circulating microparticles as disease-specific biomarkers of severity of inflammation in patients with hepatitis C or nonalcoholic steatohepatitis. Gastroenterology (2012) 143:448–58. 10.1053/j.gastro.2012.04.031 PMC3404266 22537612

[142] Welsh JA Scorletti E Clough GF Englyst NA Byrne CD Leukocyte extracellular vesicle concentration is inversely associated with liver fibrosis severity in NAFLD. J Leukoc Biol (2018) 104:631–9. 10.1002/JLB.5A1217-501R 29603349

[143] Rautou PE Bresson J Sainte-Marie Y Vion AC Paradis V Renard JM Abnormal plasma microparticles impair vasoconstrictor responses in patients with cirrhosis. Gastroenterology (2012) 143:166–76 e6. 10.1053/j.gastro.2012.03.040 22465620

[144] Thietart S Rautou PE Extracellular vesicles as biomarkers in liver diseases: A clinician’s point of view. J Hepatol (2020) 73:1507–25. 10.1016/j.jhep.2020.07.014 32682050

[145] Lambrecht J Jan Poortmans P Verhulst S Reynaert H Mannaerts I van Grunsven LA Circulating ECV-Associated miRNAs as Potential Clinical Biomarkers in Early Stage HBV and HCV Induced Liver Fibrosis. Front Pharmacol (2017) 8:56. 10.3389/fphar.2017.00056 28232800 PMC5298975

[146] Saha B Momen-Heravi F Kodys K Szabo G MicroRNA Cargo of Extracellular Vesicles from Alcohol-exposed Monocytes Signals Naive Monocytes to Differentiate into M2 Macrophages. J Biol Chem (2016) 291:149–59. 10.1074/jbc.M115.694133 PMC4697152 26527689

[147] Jiang F Chen Q Wang W Ling Y Yan Y Xia P Hepatocyte-derived extracellular vesicles promote endothelial inflammation and atherogenesis via microRNA-1. J Hepatol (2020) 72:156–66. 10.1016/j.jhep.2019.09.014 31568800

[148] Lambrecht J Verhulst S Reynaert H van Grunsven LA The miRFIB-Score: A Serological miRNA-Based Scoring Algorithm for the Diagnosis of Significant Liver Fibrosis. Cells (2019) 8:1003. 10.3390/cells8091003 PMC6770498 31470644

[149] Yang J Li C Zhang L Wang X Extracellular Vesicles as Carriers of Non-coding RNAs in Liver Diseases. Front Pharmacol (2018) 9:415. 10.3389/fphar.2018.00415 29740327 PMC5928552

[150] Lee YS Kim SY Ko E Lee JH Yi HS Yoo YJ Exosomes derived from palmitic acid-treated hepatocytes induce fibrotic activation of hepatic stellate cells. Sci Rep (2017) 7:3710. 10.1038/s41598-017-03389-2 28623272 PMC5473841

[151] Povero D Eguchi A Li H Johnson CD Papouchado BG Wree A Circulating extracellular vesicles with specific proteome and liver microRNAs are potential biomarkers for liver injury in experimental fatty liver disease. PloS One (2014) 9:e113651. 10.1371/journal.pone.0113651 25470250 PMC4254757

[152] Csak T Bala S Lippai D Kodys K Catalano D Iracheta-Vellve A MicroRNA-155 Deficiency Attenuates Liver Steatosis and Fibrosis without Reducing Inflammation in a Mouse Model of Steatohepatitis. PloS One (2015) 10:e0129251. 10.1371/journal.pone.0129251 26042593 PMC4456142

[153] Bala S Marcos M Kodys K Csak T Catalano D Mandrekar P Up-regulation of microRNA-155 in macrophages contributes to increased tumor necrosis factor {alpha} (TNF{alpha}) production via increased mRNA half-life in alcoholic liver disease. J Biol Chem (2011) 286:1436–44. 10.1074/jbc.M110.145870 PMC3020752 21062749

[154] Bala S Csak T Saha B Zatsiorsky J Kodys K Catalano D The pro-inflammatory effects of miR-155 promote liver fibrosis and alcohol-induced steatohepatitis. J Hepatol (2016) 64:1378–87. 10.1016/j.jhep.2016.01.035 PMC4874886 26867493

[155] Bala S Petrasek J Mundkur S Catalano D Levin I Ward J Circulating microRNAs in exosomes indicate hepatocyte injury and inflammation in alcoholic, drug-induced, and inflammatory liver diseases. Hepatology (2012) 56:1946–57. 10.1002/hep.25873 PMC3486954 22684891

[156] Kim HL Chung GE Park IY Choi JM Hwang SM Lee JH Elevated peripheral blood monocyte fraction in nonalcoholic fatty liver disease. Tohoku J Exp Med (2011) 223:227–33. 10.1620/tjem.223.227 21403434

[157] Wang Y Oeztuerk S Kratzer W Boehm BO Group EM-S A Nonclassical Monocyte Phenotype in Peripheral Blood is Associated with Nonalcoholic Fatty Liver Disease: A Report from an EMIL Subcohort. Horm Metab Res (2016) 48:54–61. 10.1055/s-0035-1547233 25853894

[158] Gadd VL Patel PJ Jose S Horsfall L Powell EE Irvine KM Altered Peripheral Blood Monocyte Phenotype and Function in Chronic Liver Disease: Implications for Hepatic Recruitment and Systemic Inflammation. PloS One (2016) 11:e0157771. 10.1371/journal.pone.0157771 27309850 PMC4911107

[159] Zhang J Chen W Fang L Li Q Zhang X Zhang H Increased intermediate monocyte fraction in peripheral blood is associated with nonalcoholic fatty liver disease. Wien Klin Wochenschr (2018) 130:390–7. 10.1007/s00508-018-1348-6 29845362

[160] Lefere S Puengel T Hundertmark J Penners C Frank AK Guillot A Differential effects of selective- and pan-PPAR agonists on experimental steatohepatitis and hepatic macrophages(). J Hepatol (2020) 73:757–70. 10.1016/j.jhep.2020.04.025 32360434

[161] Arias-Loste MT Iruzubieta P Puente A Ramos D Santa Cruz C Estebanez A Increased Expression Profile and Functionality of TLR6 in Peripheral Blood Mononuclear Cells and Hepatocytes of Morbidly Obese Patients with Non-Alcoholic Fatty Liver Disease. Int J Mol Sci (2016) 17:e0230307. 10.3390/ijms17111878 PMC5133878 27834919

[162] Diedrich T Kummer S Galante A Drolz A Schlicker V Lohse AW Characterization of the immune cell landscape of patients with NAFLD. PloS One (2020) 15:e0230307. 10.1371/journal.pone.0230307 32168345 PMC7069622

[163] Seike T Mizukoshi E Yamada K Okada H Kitahara M Yamashita T Fatty acid-driven modifications in T-cell profiles in non-alcoholic fatty liver disease patients. J Gastroenterol (2020) 55:701–11. 10.1007/s00535-020-01679-7 32124081

[164] Rau M Schilling AK Meertens J Hering I Weiss J Jurowich C Progression from Nonalcoholic Fatty Liver to Nonalcoholic Steatohepatitis Is Marked by a Higher Frequency of Th17 Cells in the Liver and an Increased Th17/Resting Regulatory T Cell Ratio in Peripheral Blood and in the Liver. J Immunol (2016) 196:97–105. 10.4049/jimmunol.1501175 26621860

[165] Lambrecht J van Grunsven LA Tacke F Current and emerging pharmacotherapeutic interventions for the treatment of liver fibrosis. Expert Opin Pharmacother (2020) 21:1637–50. 10.1080/14656566.2020.1774553 32543284

[166] Xia MF Yki-Jarvinen H Bian H Lin HD Yan HM Chang XX Influence of Ethnicity on the Accuracy of Non-Invasive Scores Predicting Non-Alcoholic Fatty Liver Disease. PloS One (2016) 11:e0160526. 10.1371/journal.pone.0160526 27579785 PMC5007035

[167] Bril F McPhaul MJ Caulfield MP Castille JM Poynard T Soldevila-Pico C Performance of the SteatoTest, ActiTest, NashTest and FibroTest in a multiethnic cohort of patients with type 2 diabetes mellitus. J Invest Med (2019) 67:303–11. 10.1136/jim-2018-000864 PMC6581087 30309884

[168] Bril F Millan L Kalavalapalli S McPhaul MJ Caulfield MP Martinez-Arranz I Use of a metabolomic approach to non-invasively diagnose non-alcoholic fatty liver disease in patients with type 2 diabetes mellitus. Diabetes Obes Metab (2018) 20:1702–9. 10.1111/dom.13285 29527789

[169] Arrese M Cabrera D Kalergis AM Feldstein AE Innate Immunity and Inflammation in NAFLD/NASH. Dig Dis Sci (2016) 61:1294–303. 10.1007/s10620-016-4049-x PMC4948286 26841783

[170] Lallukka S Sevastianova K Perttila J Hakkarainen A Orho-Melander M Lundbom N Adipose tissue is inflamed in NAFLD due to obesity but not in NAFLD due to genetic variation in PNPLA3. Diabetologia (2013) 56:886–92. 10.1007/s00125-013-2829-9 23334462

[171] Castera L Friedrich-Rust M Loomba R Noninvasive Assessment of Liver Disease in Patients With Nonalcoholic Fatty Liver Disease. Gastroenterology (2019) 156:1264–81 e4. 10.1053/j.gastro.2018.12.036 PMC7505052 30660725

